# Impact of carcinoid syndrome symptoms and long-term use of somatostatin analogs on quality of life in patients with carcinoid syndrome: A survey study

**DOI:** 10.1097/MD.0000000000013390

**Published:** 2018-11-21

**Authors:** Daniel M. Halperin, Lynn Huynh, Jennifer L. Beaumont, Beilei Cai, Todor Totev, Rachel H. Bhak, Mei S. Duh, Maureen P. Neary, David Cella

**Affiliations:** a Department of Gastrointestinal Medical Oncology, University of Texas MD Anderson Cancer Center, Houston, TX; b Analysis Group, Inc, Boston, MA; c Department of Medical Social Sciences, Northwestern University Feinberg School of Medicine, Chicago, IL; d Terasaki Research Institute, Los Angeles, CA; e Novartis Pharmaceuticals Corporation, East Hanover, NJ.

**Keywords:** carcinoid syndrome, neuroendocrine tumor, quality of life, somatostatin analogs

## Abstract

To evaluate association of carcinoid syndrome (CS) symptom burden and somatostatin analog (SSA) duration with quality of life (QoL) using Functional Assessment of Cancer Therapy-General (FACT-G) and Patient-Reported Outcomes Measurement Information System (PROMIS-29) instruments.

Adults who received treatment for CS symptoms in the US were recruited to participate in a cross-sectional online survey (July–October, 2016). Demographic, clinical, and QoL questions (FACT-G, 29 CS-related supplemental questions, PROMIS-29) were included. Descriptive and multivariable regression analyses adjusting for demographic and clinical characteristics followed.

Most (98%) of the 117 patients received SSAs in the prior month. Multivariable regression analysis showed ≥4 bowel movements/day (vs <4) and each additional CS symptom was associated with 7.1 (*P* = .043) and 3.4 (*P* = .034) point FACT-G total score decreases, respectively. Requiring bed rest (vs normal activity) was associated with significant decreases in FACT-G total score (*P* < .001). There were similar associations for FACT-G subscales, supplemental questions, and PROMIS-29. After adjustment, FACT-G total score was significantly higher (11.3 points; *P* = .033) for patients treated with SSA >8 years versus <2.7 years.

CS symptom burden was observed to be associated with lower QoL scores, measured by FACT-G. Patients with >8 years SSA treatment duration versus <2.7 years had higher QoL.

## Abstract 2

Supplemental Digital Content is available in the text

## 1 Introduction

Neuroendocrine tumors (NETs) are malignancies that arise from a diffuse network of neuroendocrine cells throughout the body. Carcinoid syndrome (CS) results from secretion of bioactive amines, peptides, and polypeptides by functional NETs. Symptoms may include diarrhea, flushing, wheezing, and less frequently carcinoid heart disease, characterized by cyanosis and/or peripheral edema.^[1,2]^

Among patients with NET in the United States (US) identified between 2000 and 2011 in the Surveillance, Epidemiology, and End Results (SEER) data of the National Cancer Institute, 19% were found to have CS at diagnosis with common subgroups such as metastatic well-differentiated small bowel NET manifesting CS in over 50% of cases.^[1]^ First-line systemic therapy for metastatic NETs frequently includes somatostatin analogs (SSAs) such as octreotide or lanreotide which inhibit the secretion of gastrointestinal hormones.^[3]^ Octreotide, approved in 1988, is indicated for the treatment of CS arising from metastatic NETs, including severe diarrhea and flushing.^[4]^ It has also been demonstrated to improve progression-free survival in patients with advanced well-differentiated midgut NETs.^[5]^ Lanreotide was approved in 2014 for the treatment of patients with unresectable, well- or moderately-differentiated, locally advanced or metastatic gastroenteropancreatic NETs based on randomized trial data demonstrating improved progression-free survival.^[6]^

Prior studies have demonstrated that patients with NET and CS often report reduced quality of life (QoL) relative to the general population, particularly in areas of fatigue, general health, and physical role limitations.^[7–9]^ Frequent bowel movements and flushing episodes have been shown to be significantly associated with decreased health-related QoL.^[10]^ The objectives of this study were to use the Functional Assessment of Cancer Therapy-General (FACT-G) and Patient-Reported Outcomes Measurement Information System (PROMIS)-29 instruments to assess the association between the burden of CS symptoms and QoL among patients with CS, and to assess the association between the duration of SSA use and QoL among patients with CS symptoms. The QoL assessment also included 29 CS symptom-specific supplemental questions to the FACT-G.

## 2 Materials and methods

### 2.1 Data source and eligibility

Patients with CS symptoms in the US were recruited through the Neuroendocrine Cancer Awareness Network (NCAN), a patient advocacy group, for participation in an online, anonymous cross-sectional survey between July 21 and October 31, 2016. An email message with a description of the survey was sent to NCAN members expected to be patients with NET, and interested persons were able to access the survey through a Web-based electronic data capture platform. Patients were told the survey would require approximately 30 minutes, and they would be given a gift card of nominal value for completing the survey. To prevent multiple survey completions or link sharing, single-use survey links were used in the email recruitment.

Patients qualified to participate in the survey if they were at least 18 years old at the time of taking the survey, diagnosed with NET and CS by a physician, and were treated with either SSA or non-SSA treatments for CS symptom control. SSA treatment that patients reported included lanreotide, octreotide, and pasireotide. Non-SSAs included cyproheptadine, diphenoxylate-atropine, diphenhydramine, loperamide, ranitidine, and telotristat etiprate.

The survey consisted of demographic characteristics (e.g., gender, age, race), clinical characteristics (e.g., site of NET, time since NET and CS diagnoses, treatments, CS symptoms such as flushing episodes, bowel movements, and their frequency and severity, and activity levels), and QoL questions from the validated FACT-G (Version 4)^[11,12]^ and PROMIS-29 Profile v2.0^[13,14]^ questionnaires. The FACT-G is a 27-item questionnaire divided into 4 primary QoL domains (Physical Well-Being [PWB], Social Well-Being [SWB], Emotional Well-Being [EWB], and Functional Well-Being [FWB]) to ascertain well-being in the past 7 days. All questions about well-being use a 5-point rating scale (0 = “Not at all;” 1 = “A little bit;” 2 = “Somewhat;” 3 = “Quite a bit;” 4 = “Very much”). Questions that have negative wording (e.g., “I have pain”) use a reverse scoring system so that a higher score is associated with better QoL^[15]^. Twenty-nine supplemental questions from the FACT-G item library were also included. The questions were selected from the Functional Assessment of Chronic Illness Therapy (FACIT) item library specifically for their relevance to QoL of patients with CS and were reviewed by clinical and QoL experts. Patients were required to complete all questions before moving on to the next question to ensure no missing responses.

The PROMIS-29 instrument covers 7 domains (Depression, Anxiety, Physical Function, Pain Interference, Fatigue, Sleep Disturbance, and Ability to Participate in Social Roles and Activities) with 4 questions per domain. All domains are assessed for the past 7 days, except for Physical Function which has no time frame. Questions within the domains have 5 response options. For symptom domains, higher scores indicate worse outcomes, and on function domains, lower scores indicate worse outcomes. There is also a pain intensity numerical rating scale from 0 to 10.^[16]^

Data were deidentified and complied with the patient confidentiality requirements of the Health Insurance Portability and Accountability Act (HIPAA). All study materials were approved by the New England Independent Review Board. Patients provided their informed consent prior to responding to the survey questions.

### 2.2 Statistical analyses

Descriptive analyses assessing patient demographic and clinical characteristics and NET and CS treatment characteristics were conducted using means and standard deviations (SDs) for continuous variables and frequencies and proportions for categorical variables.

For the FACT-G survey, subscale scores were computed for each of the 4 primary QoL domains (PWB, SWB, EWB, FWB). Item responses for each domain were summed to compute the subscale score for each domain according to previously published scoring algorithms.^[15]^ The total score was computed by summing subscale scores. Means and SD of scores were reported. Similarly, a total score for the supplemental questions was calculated as the sum of the scores across all 29 attributes. Means and SD of scores for the total and individual FACT-G supplemental questions were also reported.

For the PROMIS-29 survey, a raw score for each of the 7 domains was calculated. The responses for the questions within the domain were summed to compute the raw score for the domain according to published methods.^[16]^ This score was rescaled into a T-score which is standardized to a mean of 50 and a SD of 10; thus, a T-score of 40 is equivalent to being 1 SD below the mean of the US population.^[16]^

There were no missing responses to the FACT-G and PROMIS-29 questions, so pro-rating of scores was not necessary.

Multivariable linear regression analyses, adjusting for demographic (gender, age, and race) and clinical characteristics (carcinoid/NET type, number or frequency of symptoms, and current activity level), were performed to assess the association between CS symptoms and the outcomes of total FACT-G score, FACT-G subscales, and PROMIS-29 domain T-scores. To assess the association between CS burden and QoL and to avoid multicollinearity due to the correlation between the various CS burden questions, 2 multivariable models were used. In the first model, CS burden was assessed by the average number of bowel movements and flushing episodes per day. In the second model, CS burden was assessed by the total number of symptoms experienced. To assess the association between duration of SSA use and QoL, duration of SSA use was categorized into quartiles (<2.7 years, 2.7–4.42 years, 4.43–8.0 years, and >8.0 years). A sensitivity analysis using FACT-G data was conducted using a multilevel hierarchical model to adjust for the nested effect of duration of SSA use within duration of time since CS diagnosis; level 1 included SSA duration and patient characteristics, while time since CS diagnosis was treated as a random effect in level 2. For all regression analyses, statistically significant associations were indicated by *P* < .05.

In addition, a Pearson correlation coefficient matrix was calculated for a subset of the supplemental FACT-G questions to determine questions that appeared to be associated with each other, and those with significant correlation (*P* < .05) were grouped into subsequently identified thematic sets (diarrhea, dyspnea, flushing, rash, cognitive ability, other attributes). Principal component analysis with rotated factor loadings using the varimax method was used to confirm the groupings that were identified within the supplemental questions. For each thematic set, a mean and SD were reported.

All analyses were performed using SAS software version 9.4 (SAS Institute, Inc, Cary, NC).

## 3 Results

### 3.1 Demographic, clinical, and treatment characteristics

Of the 117 patients who completed the online survey, the majority were female (77%) and Caucasian (91%), with a mean age of 58.0 years (Table 1). The most common primary site of NET was ileum (47%), and the mean (SD) time from NET diagnosis to date of survey completion was 8.3 (6.0) years. The mean time from NET diagnosis to diagnosis of CS was 1.2 years. The most frequently reported CS symptoms among the 6 different symptoms queried in the survey included diarrhea (97%), flushing (91%), peripheral edema (46%), and wheezing (41%). Forty-five percent of the patients reported experiencing at least 4 bowel movements daily, and 12% experienced at least 4 flushing episodes per day in the past 2 weeks. At the time of the survey, 86% reported bed rest less than 50% of the waking day.

**Table 1:**
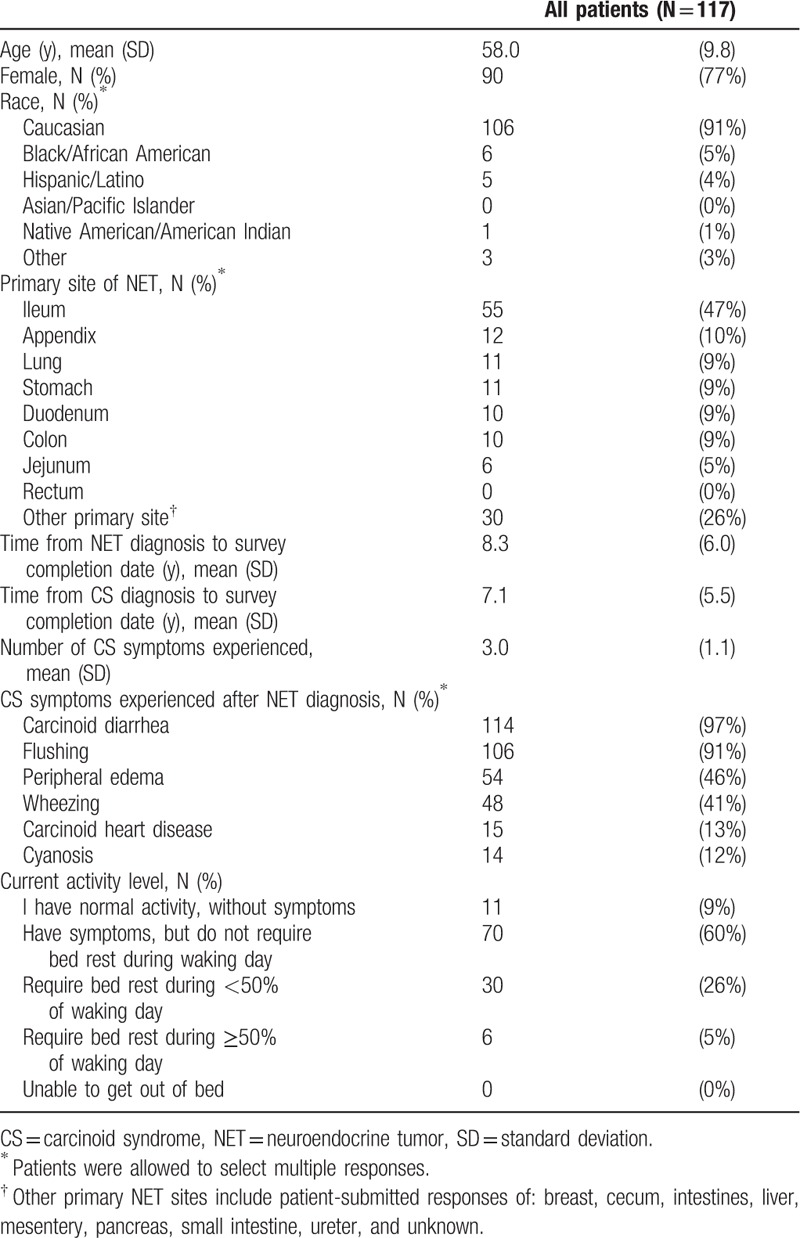
Demographic and clinical characteristics of survey patients with carcinoid syndrome.

Most frequently, patients reported ever undergoing treatment with SSAs (94%), surgical procedures (72%), and liver-directed therapy (35%) (Table 2). For CS symptom treatment, the large majority of patients reported receiving SSAs in the past month (98%). The median duration of CS symptom treatment with SSAs was 4.4 (range: 0.3–21.3) years. Forty-three percent of patients treated with SSAs reported receiving SSA treatment for longer than 5 years. Most patients were still receiving CS symptom treatment with SSAs (97%) at the time of the survey. The most commonly reported SSA treatment adjustment was increased total monthly dose of SSA (58%), while 29% of patients did not have any treatment adjustment. The most frequently given reason for prescription of SSA for CS symptom control was that it was standard treatment (90%), followed by the demonstrated efficacy (43%), and tolerability (33%).

**Table 2:**
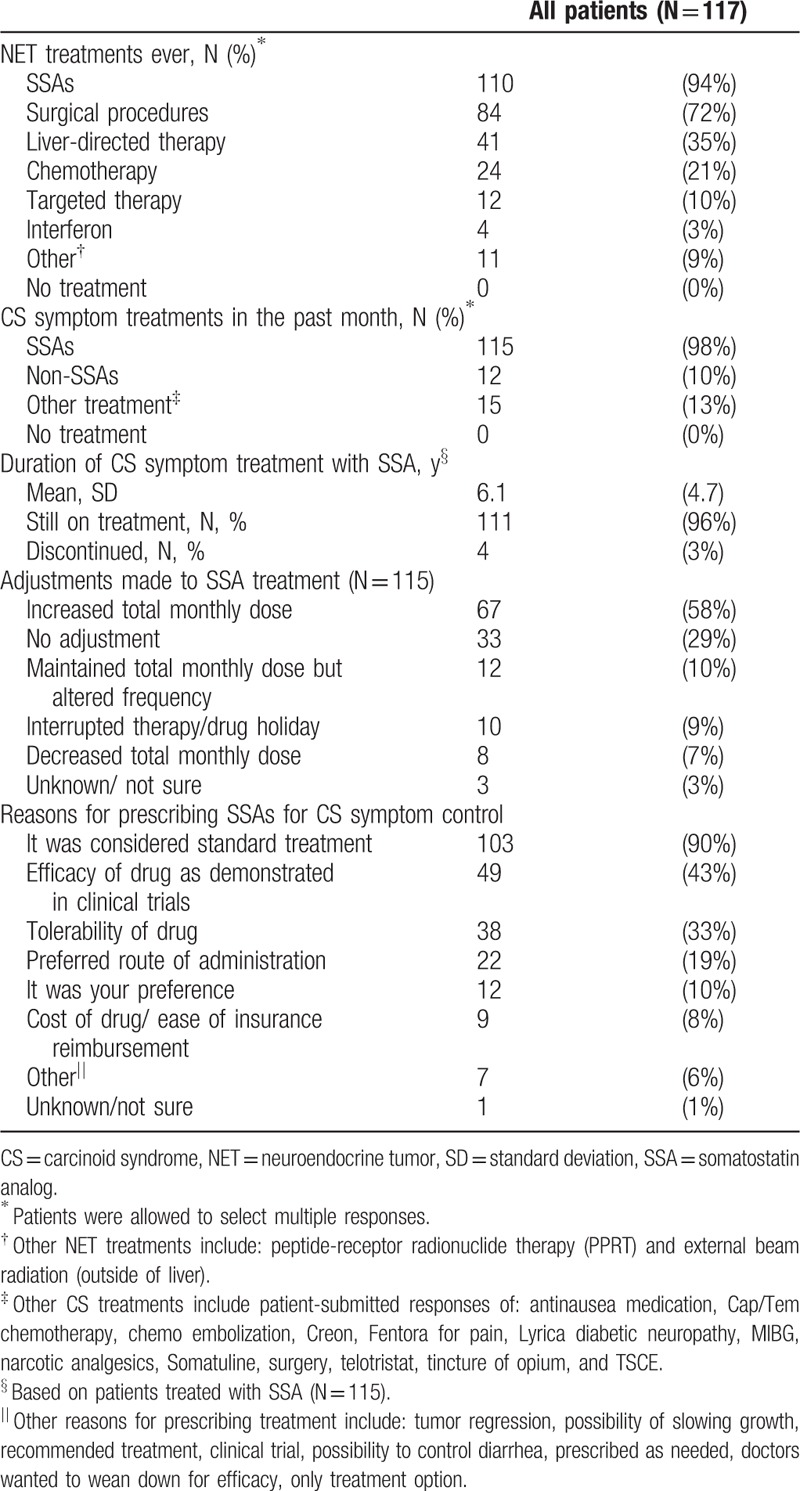
Neuroendocrine tumor and carcinoid syndrome treatment characteristics of survey patients with carcinoid syndrome.

### 3.2 FACT-G and supplemental questions

**Figure 1:**
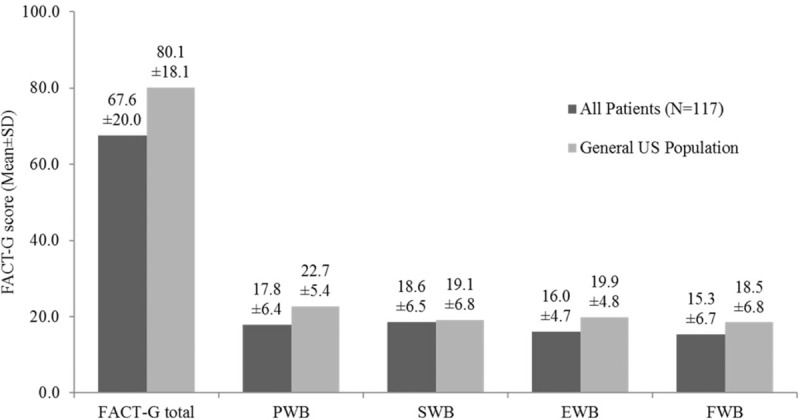
FACT-G scores of survey patients with carcinoid syndrome versus US general population.^[1]^ EWB = emotional well-being, FACT-G = Functional Assessment of Cancer Therapy-General, FWB = functional well-being, PWB = physical well-being, SD = standard deviation, SWB = social well-being, US = United States. [1] General US population scores as reported in Webster et al ^[15]^

The mean (SD) FACT-G total score was 67.6 (20.0), which is lower than the general US population score of 80.1 (18.1)^[15]^; PWB, SWB, EWB, and FWB mean scores were also lower than those of the general US population (Fig. 1). The mean (SD) total score from the supplemental questions to the FACT-G was 65.7 (20.4). Multivariable regression analysis showed that the level of severity of CS symptoms experienced was associated with reduced QoL scores in the analysis of FACT-G data. Having at least 4 bowel movements per day was associated with a 7.1 point lower FACT-G total score than patients with fewer than 4 bowel movements per day (*P* = .043); similar associations were observed in the subscales and the total score of the supplemental questions (Table 3). For each additional CS symptom experienced, the FACT-G total score decreased by 3.4 points (*P* = .034). Similarly, the PWB, SWB, EWB, and FWB scores were also reduced by 0.6 to 1.1 points and by 5.0 points for the total score of the supplemental questions (Table 4). Reduced activity levels (i.e., some symptoms but no bed rest, bed rest for <50% of the day or ≥50% of the day compared with normal activity) were also associated with decreases in the FACT-G total score. This trend was also seen in PWB, SWB, EWB, and FWB subscales, and the total score of the supplemental questions (Tables 3 and 4). After adjustment, the FACT-G total score was significantly higher (better) (11.3 points; *P* = .033) for patients treated with SSA more than 8 years (4th quartile) compared with those treated with SSA for less than 2.7 years (1st quartile) but not compared with those treated with SSA for intermediate durations of treatment. Similar associations were observed for 2 FACT-G subscales—PWB and FWB (Table 5). The scores for the SWB and EWB subscales and the total score of the supplemental questions were higher, but not statistically significantly, for patients treated with SSA more than 8 years versus less than 2.7 years. In the sensitivity analysis that treated time since CS diagnosis as a random effect, the hierarchal model showed patients treated with SSAs for more than 8 years had significantly higher FACT-G total scores and PWB, EWB, and FWB subscale scores compared with patients treated with SSAs for less than 2.7 years (by 11.7 points; *P* = .017). The association between treatment duration and the SWB subscale and the FACT-G supplemental total scores was positive, but not statistically significant, using the hierarchical model.

**Table 3:**
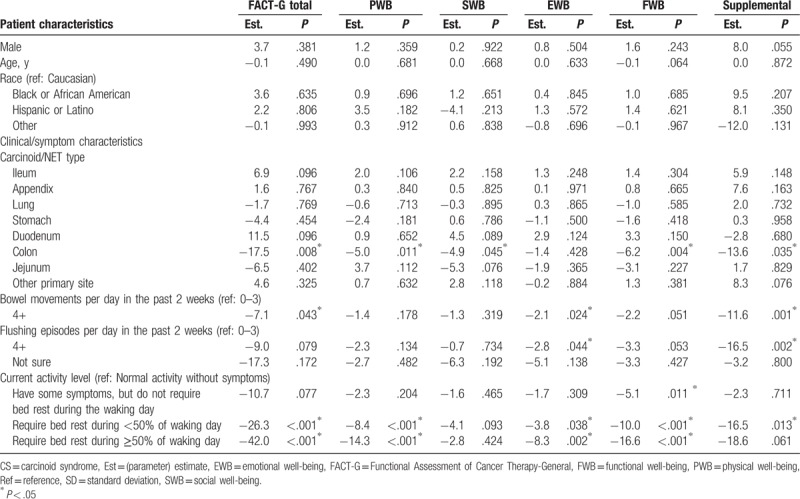
Multivariable predictors of FACT-G scores: carcinoid syndrome symptoms as measured by bowel movements and flushing episodes (Model 1).

**Table 4:**
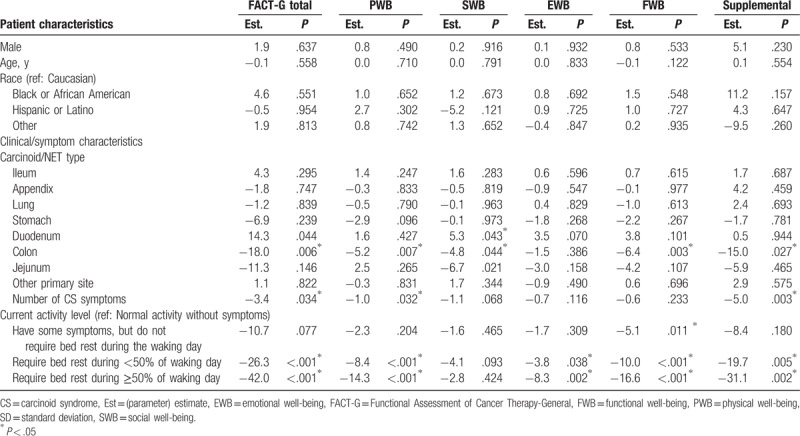
Multivariable predictors of FACT-G scores: carcinoid syndrome symptoms as measured by total number experienced (Model 2).

**Table 5:**
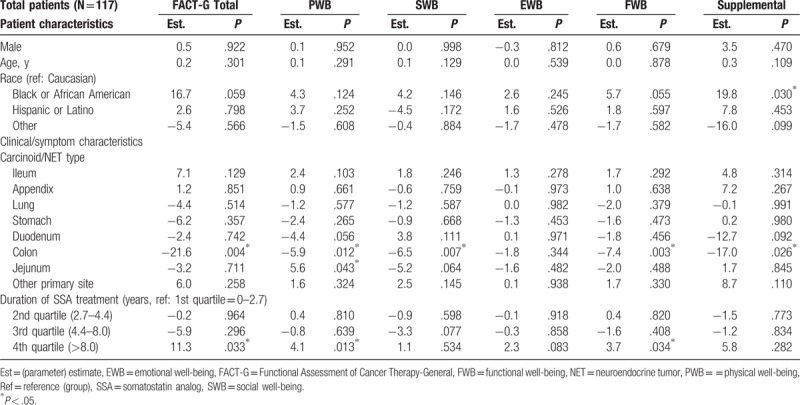
Multivariable predictors of FACT-G scores: somatostatin analog treatment duration.

The results from the Pearson correlation coefficient matrix of supplemental questions to FACT-G showed significant correlations among sets of attributes. This informed the grouping of 18 of the attributes by common themes: diarrhea, dyspnea, flushing, rash, and cognitive ability. The remaining 11 attributes were reported separately. A principal components analysis with varimax rotated factor loadings was conducted on all attributes asked on the “not at all” to “very much” scale (these excluded the attributes related to cognitive ability, which were asked on the “never” to “several times a day” scale). This analysis confirmed the thematic sets previously identified through correlations; the attributes within each set had eigenvalues ≥1 and factor loadings >.50. Quality of life scores for the supplemental questions to FACT-G, along with the total score and the summary scores for the thematic sets are reported in Supplemental Table 1.

### 3.3 PROMIS-29

**Figure 2:**
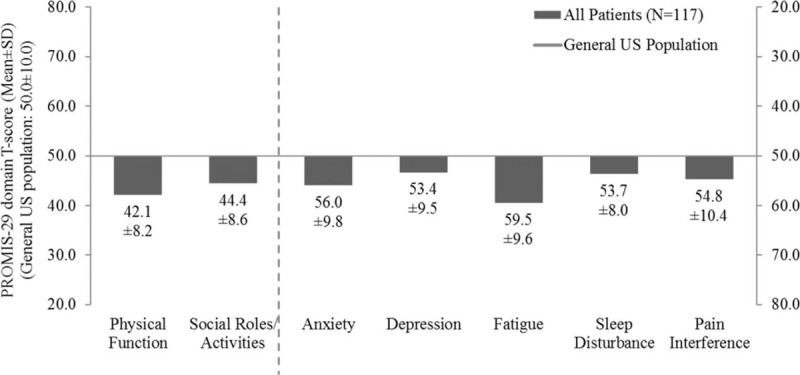
PROMIS-29 scores of survey patients with carcinoid syndrome versus US general population.^[1]^ PROMIS = Patient-Reported Outcomes Measurement Information System, SD = standard deviation, US = United States. [1] T-scores reported standardized, where a score of 50 is the average for the US general population.^[16]^

The mean (SD) T-score for physical function and fatigue were 42.1 (8.2) and 59.5 (9.6), respectively, which were both about 1 SD worse than the general US population score of 50.0 (10.0);^[16]^ the 5 other domains were also worse than the general US population: social role (44.4 [8.6]), anxiety (56.0 [9.8]), depression (53.4 [9.5]), pain interference (54.8 [10.4]), sleep disturbance (53.7 [8.0]) (Fig. 2). Multivariable regression analysis showed that the severity of CS symptoms experienced was associated with reduced QoL scores in the analysis of PROMIS-29 data. More than 4 bowel movements per day was significantly associated with a 3.0 point decrease in the score for Ability to Participate in Social Roles and Activities as compared with having less than 4 bowel movements per day (*P* = .043) (Table 6). Similarly, the multivariable model with number of CS symptoms showed that each additional CS symptom experienced significantly worsened Physical Function, Ability to Participate in Social Roles and Activities, Fatigue, and Pain Interference (by magnitudes of 1.6–1.9 points in PROMIS-29 T-scores, *P* ranged from .004 to .029) (Table 7). Requiring any amount of bed rest during the day (compared with normal activity without CS symptoms) was also associated with decreases in the PROMIS-29 domain scores, depending on activity level (Tables 6 and 7). The length of SSA treatment duration was not found to be a significant predictor of any PROMIS-29 domain T-scores, after adjustment for potential confounding factors (Table 8).

**Table 6:**
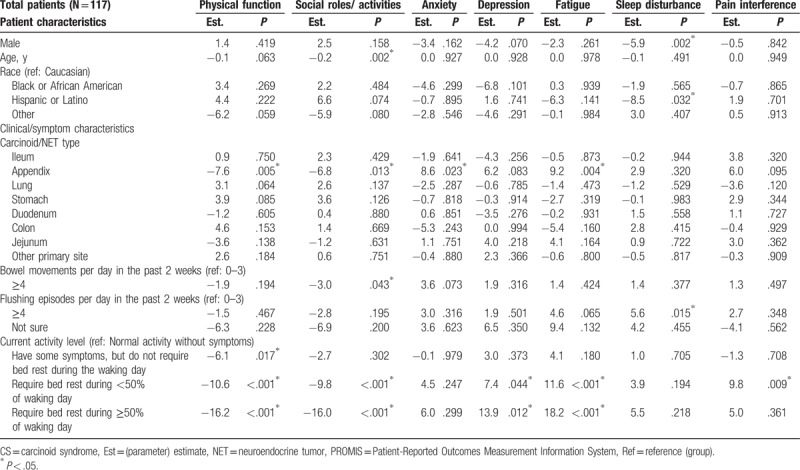
Multivariable predictors of PROMIS-29 scores: carcinoid syndrome symptoms as measured by bowel movements and flushing episodes (Model 1).

**Table 7:**
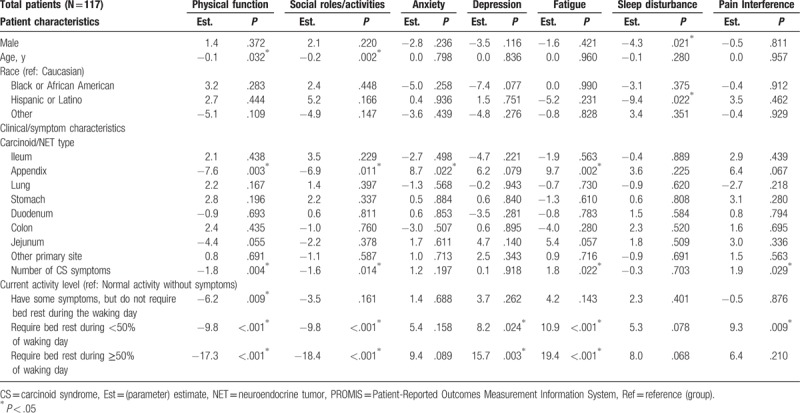
Multivariable predictors of PROMIS-29 scores: carcinoid syndrome symptoms as measured by total number experienced (Model 2).

**Table 8:**
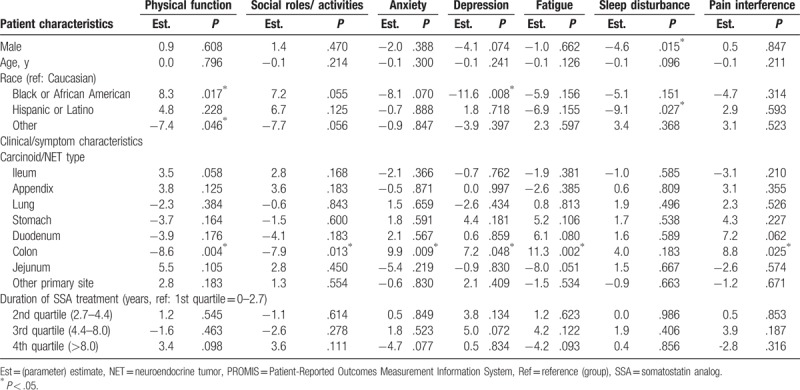
Multivariable predictors of PROMIS-29 scores: somatostatin analog treatment duration.

## 4 Discussion

This study found that CS symptom burden as captured by more frequent flushing and bowel movements, as well as a greater number of symptoms experienced and impaired activity levels, was associated with lower QoL, as demonstrated by FACT-G and FACT-G supplemental questions, and PROMIS-29, among patients with CS who are often treated with SSAs. QoL as measured by both instruments was lower for patients with CS as compared with data previously collected from the general US population. Patients treated with SSAs for longer than 8 years had significantly higher (better) FACT-G total score and PWB and FWB subscale scores when compared with patients treated with SSAs for fewer than 2.7 years.

The finding that a greater number of bowel movements and flushing episodes was associated with worse QoL is consistent with the published literature.^[7,10]^ Using the PROMIS-29 instrument, Beaumont et al ^[7]^ and Pearman et al ^[10]^ showed a pronounced effect of bowel movement and flushing on QoL when patients experienced 4 or more bowel movements per day and 1 or more flushing episodes in a 2-week period. The findings in the current and prior studies are important as they demonstrate that even a low frequency of an event like flushing, which might be dismissed by a clinician, can have a pronounced effect on QoL. The population in the current study had similar demographics in terms of age and gender to those included in Beaumont et al ^[7]^ and Pearman et al ^[10]^ and were recruited by a similar mechanism, though there were additional inclusion criteria requiring patients have CS and treatment. These studies also had similar rates for patients experiencing 4 or more bowel movements per day and 4 or more flushing episodes per day. Results presented here are consistent with the existing literature on the relevance of high frequency bowel movement and flushing to QoL as assessed by 2 validated instruments and a multivariable analysis.

Prior studies with other QoL instruments and study populations have also shown that patients with NETs have significantly lower QoL relative to the general population; the difference in QoL in the current study's population of NET patients and the general population is greater than those reported in other studies, however. Patients with NETs were reported to have lower QoL than the general population in another study in the US when assessed with the SF-36 and PROMIS-29.^[7]^ While the PROMIS-29 scores were worse than those in the other US study, the results were consistent by showing that among the PROMIS-29 scales, physical function and fatigue scores deviated the most from the general population.^[7]^ The mean (SD) physical function and fatigue scores in Beaumont et al^[7]^ versus those presented here were 45.0 (9.3) versus 42.1 (8.2) and 54.7 (10.6) versus 59.5 (9.6), respectively. Notably, the NET patients in the US study by Beaumont et al^[7]^ included any patient diagnosed with NET; 65% had CS and 47% had not experienced flushing episodes in the past 2 weeks. In contrast, the current study required participants to have NET and CS, and to be treated for CS; only 22% had not experienced flushing episodes in the past 2 weeks. A study among NET patients who were treated in Norway (but not necessarily with CS) was conducted by Haugland et al^[8]^ using the SF-36. They reported significantly worse measures for all scales except bodily pain for the NET patients relative to the general population.^[8]^ Similar to these results, the role-physical limitation subscale exhibited the largest difference between NET patients and the general population in the Norwegian study, with the mean score being 25 points lower among NET patients; the reported effect size for role-physical limitation in the Norwegian study for the patient population relative to the general population was 0.57.^[8]^ Frojd et al^[9]^ conducted a longitudinal study of QoL with the EORTC QLQ-C30 among NET patients in Sweden and also reported significant differences between NET patients and population norms; NET patients scored 10 points below population norms for role function, social function, and global QoL and 10 points above for fatigue and diarrhea. Unlike the current analysis, the study conducted in Sweden did not require patients to have CS, and 44% of the patients in the Swedish study had no NET treatment. ^[9]^ The phase 3, multicenter, RADIANT-4 trial randomized patients to everolimus or placebo for treatment of advanced, nonfunctional NETs of lung or GI origin and reported mean FACT-G scores at baseline that were not very different from the US general population; the mean (SD) total FACT-G score for patients in the everolimus treatment arm was 81.2 (15.5) versus 80.1 (18.1) in the US general population.^[15,17]^ However, among NET patients in the trial, pre- and postprogression FACT-G scores were significantly different. Relative to the trial population, this study population may have had worse QoL as they had functional NETs and were included regardless of symptoms, disease stage, and prior treatment. In addition, there may be differences between patients and their disease severity and QoL for those who seek participation in a trial versus those joining a patient advocacy group.

Almost all patients (98%) taking part in this survey study reported using SSAs for CS symptom control. The mean duration of SSA use was 6.1 years, and the median was 4.4 years. Possible explanations as to why a greater percentage of patients reported SSA use for treatment of CS versus treatment of NET (98% vs 94%, respectively) include that patients understood SSA to be prescribed for CS symptom control only, or that clinicians continued SSA therapy following tumor progression to control symptoms while using additional agents for tumor control.

The association between improved QoL measured by FACT-G and SSA treatment greater than 8 years versus SSA treatment less than 2.7 years may in part be explained by long-term effectiveness and tolerability of SSAs. However, there is also the possibility that selection bias favoring patients with more indolent disease (in the longer duration group) may have had some effect on the results. Certain FACT-G subscales were more sensitive to SSA treatment duration in CS patients compared with PROMIS-29. This may be due to FACT-G's PWB and FWB subscales containing disease- and treatment-specific items whereas PROMIS-29's general QoL attributes were designed for a wide range of chronic diseases. The multivariable analysis results were robust after performing sensitivity analysis using a hierarchical model to adjust for the nested effect of duration of SSA use within duration of time since CS diagnosis. To our knowledge, this is the first study to examine the relationship between SSA treatment duration and QoL. Beaumont et al^[7]^ reported that SSA use was not significantly associated with QoL in multivariable models of SF-36 and PROMIS-29 subscales, and Pearman et al^[10]^ compared PROMIS-29 scores between patients receiving different treatments, but neither study examined SSA treatment duration specifically.

QoL has become an important endpoint for evaluating patients’ well-being, particularly in the case of oncology where therapeutic interventions may have associated toxicities. QoL assessments provide input from the patient perspective and add to the understanding of the measured effects of treatments which are generally physician-collected outcomes.^[18]^ Previously published clinical trials have shown that treatment of NETs with SSAs and sunitinib is associated with clinical benefit such as increase in time to tumor progression and survival relative to placebo or best standard of care while not compromising QoL in the treated populations.^[5,19,20]^ A small crossover trial following the development of SSAs demonstrated that octreotide relieved GEP-NET symptoms and restored QoL.^[21]^ QoL data from patients treated in real world settings are important for evaluating the full impact of treatment on patient lives, decision making about treatment options, and improving the allocation of healthcare resources.^[22]^ Controlling hormonal secretion may also be particularly important for NET patients as CS is associated with worse overall survival.^[1]^

This study is subject to some limitations. First, potential survival bias may exist as patients who lived with CS longer may be healthier and have better QoL regardless of their treatment. A sensitivity analysis was performed to include both SSA treatment duration and duration of time since CS diagnosis in a hierarchical model to adjust for the nested effect, and the same trends were seen. Additionally, all data were self-reported and could have been subject to recall bias; furthermore, limited clinical data were collected. This was a cross-sectional and observational study, and results are based on QoL assessment at a single time point. Lastly, recruitment was conducted primarily through the patient support organization NCAN, which is likely a biased sample, not fully representative of the heterogeneous NET patient population.

In conclusion, this study suggests that increased CS symptom burden, as measured by higher frequency of flushing and bowel movements, greater number of symptoms and impaired activity levels, is associated with lower QoL. Patients with CS were observed to be often treated with SSAs, while those treated with SSAs for more than 8 years were observed to have higher QoL compared with those treated with SSAs for less than 2.7 years.

## Acknowledgments

The authors thank the patients, families, and their caregivers for participating in this survey, as well as Maryann and Robert Wahmann from NCAN, for their collaboration with the survey outreach. The authors thank Caroline Korves, ScD of Analysis Group, Inc for her assistance with developing this manuscript.

## Author contributions

**Conceptualization:** Daniel M Halperin, Jennifer Beaumont, Mei S Duh, Maureen P Neary, David Cella.

**Data curation:** Daniel M Halperin.

**Formal analysis:** Lynn Huynh, Todor Totev, Rachel H Bhak, David Cella.

**Funding acquisition:** Beilei Cai, Maureen P Neary.

**Investigation:** Daniel M Halperin, Jennifer Beaumont, Beilei Cai, Rachel H Bhak.

**Methodology:** Daniel M Halperin, Lynn Huynh, Jennifer Beaumont, Beilei Cai, Todor Totev, Mei S Duh, Maureen P Neary, David Cella.

**Project administration:** Lynn Huynh, Todor Totev, Rachel H Bhak, Mei S Duh.

**Software:** Lynn Huynh.

**Supervision:** Lynn Huynh, Beilei Cai, Mei S Duh, Maureen P Neary.

**Validation:** Jennifer Beaumont.

**Writing – original draft:** Lynn Huynh.

**Writing – review & editing:** Daniel M Halperin, Lynn Huynh, Jennifer Beaumont, Beilei Cai, Todor Totev, Rachel H Bhak, Mei S Duh, Maureen P Neary, David Cella.

## Supplementary Material

Supplemental Digital Content
