# Supplementary material for: Impact of carcinoid syndrome symptoms and long-term use of somatostatin analogs on quality of life in patients with carcinoid syndrome: A survey study
Source: Medicine (Baltimore). 2018 Nov 21;97(47):e13390. doi: 10.1097/MD.0000000000013390 (PMC6392719; doi:10.1097/MD.0000000000013390)
Supplement: Supplemental Digital Content [file medi-97-e13390-s001.docx]

#### Supplemental Table 1. Quality of Life Scores from Supplemental Questions to FACT-G, Grouped by Thematic Sets

|  | All Patients | |
| --- | --- | --- |
|  | (N=117) | |
| **Total**^1^, mean (SD) | 65.7 | (20.4) |
| **Diarrhea**, mean (SD) | 2.3 | (1.1) |
| My problem with diarrhea keeps/wakes me up at night | 2.8 | (1.2) |
| I have to limit my social activity because of diarrhea | 2.4 | (1.3) |
| I have to limit by activities due to diarrhea | 2.3 | (1.3) |
| I am afraid to be far from a toilet | 2.2 | (1.3) |
| I have abdominal cramps or discomfort (due to my diarrhea) | 2.2 | (1.3) |
| I have diarrhea | 1.7 | (1.0) |
| **Dyspnea**, mean (SD) | 2.6 | (1.3) |
| I have been short of breath | 2.4 | (1.3) |
| At times I feel breathless | 2.7 | (1.4) |
| **Flushing**, mean (SD) | 2.6 | (1.0) |
| I am bothered by flushing episodes | 2.7 | (1.2) |
| I have flushing episodes | 2.5 | (1.2) |
| I have hot flushes | 2.5 | (1.2) |
| **Rash**, mean (SD) | 3.0 | (1.1) |
| I am bothered by a skin rash | 3.3 | (1.1) |
| I have a rash or skin reddening on my face, neck or chest | 3.0 | (1.3) |
| I am bothered by itching | 2.8 | (1.4) |
| **Cognitive ability**, mean (SD) | 2.0 | (1.3) |
| I have trouble thinking clearly | 2.2 | (1.4) |
| My thinking has been slow | 2.1 | (1.3) |
| I have had trouble concentrating | 2.0 | (1.3) |
| I have trouble remembering information | 1.8 | (1.2) |
| **Other attributes, not grouped by theme**, mean (SD) | - | - |
| I have had a problem from repeated injections | 3.2 | (1.1) |
| I have had side-effects from my treatment | 2.5 | (1.3) |
| I am bothered by a change in weight | 2.5 | (1.3) |
| I have discomfort or pain in my stomach area | 2.1 | (1.3) |
| I feel bloated | 2.0 | (1.3) |
| I am able to do my usual activities^2^ | 2.0 | (1.2) |
| I am able to eat the foods that I like^2^ | 1.9 | (1.1) |
| I worry about the effect of stress on my illness | 1.9 | (1.2) |
| I have to take my condition into account when making plans | 1.5 | (1.3) |
| Certain foods or drinks can make my symptoms worse | 1.2 | (1.1) |
| Physical or emotional stress can make my symptoms worse | 1.2 | (1.2) |

SD, Standard Deviation.
[1] Total score was calculated as the sum of the scores across all 29 attributes. The possible range was from 0-116.

[2] Question reverse-scored for contextual consistency with the rest of the attributes.
